# Extracellular Vesicles: Schistosomal Long-Range Precise Weapon to Manipulate the Immune Response

**DOI:** 10.3389/fcimb.2021.649480

**Published:** 2021-03-18

**Authors:** Dror Avni, Orly Avni

**Affiliations:** ^1^ Laboratory of Molecular Cell Biology, Sheba Medical Center, Tel Hashomer, Israel; ^2^ Laboratory for the Study of Tropical Diseases, Sheba Medical Center, Tel Hashomer, Israel; ^3^ Department of Medicine C, Sheba Medical Center, Tel Hashomer, Israel; ^4^ The Azrieli Faculty of Medicine, Bar-Ilan University, Safed, Israel

**Keywords:** extracellular vesicles, *Schistosoma*, Th2 immunity, miRNAs, M1 pathway

## Abstract

Schistosomiasis (Bilharziasis), a neglected tropical disease that affects more than 240 million people around the world, is caused by infection with the helminth parasite *Schistosoma*. As part of their secretome, schistosomes release extracellular vesicles (EVs) that modulate the host immune response. The EV-harbored miRNAs upregulate the innate immune response of the M1 pathway and downregulate the differentiation toward the adaptive Th2 immunity. A schistosomal egg-derived miRNA increases the percentage of regulatory T cells. This schistosomal-inducible immunoediting process generates ultimately a parasitic friendly environment that is applied carefully as restrained Th2 response is crucial for the host survival and successful excretion of the eggs. Evidence indicates a selective targeting of schistosomal EVs, however, the underlying mechanisms are unclear yet. The effects of the schistosomes on the host immune system is in accordance with the hygiene hypothesis, attributing the dramatic increase in recent decades in allergy and other diseases associated with imbalanced immune response, to the reduced exposure to infectious agents that co-evolved with humans during evolution. Deciphering the bioactive cargo, function, and selective targeting of the parasite-secreted EVs may facilitate the development of novel tools for diagnostics and delivered therapy to schistosomiasis, as well as to immune-associated disorders.

## Introduction

Schistosomiasis is caused by the trematode helminth of the genus *Schistosoma*. The three main species that infect humans, the definite hosts, are *S. mansoni* (Africa, South America, Caribbean, and Middle East), *S. japonicum* (China and South East Asia), and *S. haematobium* (Africa and Middle East) (McManus et al., 2018). Humans are infected in freshwater bodies, where the schistosomal cercariae penetrate the skin, transform into schistosomula that migrate to the lungs, and then to the liver. In the liver, the male and female copulate and mature into adult worms that further migrate together to their final destination–either urogenital venules for *S. haematobium* or mesenteric venules for *S. mansoni* and *S. japonicum* (Gryseels, 2012). In the venules, the female produces eggs that reach the water through either urine or feces. The miracidia larvae that hatch from these eggs invade specific aquatic snails, the intermediate hosts, in which asexual reproduction yields thousands of infective cercariae (Dunne and Cooke, 2005). Symptoms of schistosomiasis are caused mostly by the immune response to eggs that failed to be delivered out of the body and become trapped in tissues, where they may induce intestinal, hepato-splenic or urogenital diseases (McManus et al., 2018).

The infection course of schistosomiasis and the immune response of the host evolved in concert during continuing coevolutionary relationship that exerts selective pressures on the parasite for survival and reproduction without excessively harming its host, and on the host to expel the invasive parasite with a minimum collateral damage to its own tissues (Allen and Maizels, 2011). Parasites release biologically active molecules, and it is clear now that some of them are packed inside extracellular vesicles (EVs) (Coakley et al., 2015; Siles-Lucas et al., 2015; Eichenberger et al., 2018; Zakeri et al., 2018; Ofir-Birin and Regev-Rudzki, 2019; Bischofsberger et al., 2020). EVs are cell-derived membrane-enclosed particles that varied by their size, content, and intra-cellular origin. It was demonstrated that both *S. japonicum* and *S. mansoni* adult worms, as well as *S. mansoni* schistosomula and *S. japonicum* eggs, secrete EVs (Wang et al., 2004; Nowacki et al., 2015; Sotillo et al., 2016; Zhu et al., 2016a; Zhu et al., 2016b; Samoil et al., 2018; Kuipers et al., 2020; Meningher et al., 2020). This review is focused on the current knowledge on the way by which schistosomal EVs modulate the immune response, mostly as was learned from the murine model. However, these findings are applicable to humans, as schistosomal microRNAs (miRNAs) have been found in blood-derived EVs of schistosomal infected humans (Meningher et al., 2017). Deeper dissection of these communicable particles may facilitate the development of novel approaches to treat schistosomiasis and retuning immune disorders.

### Schistosomal EVs Promote the Innate M1 Immune Response

It was found that adult *S. japonicum*-derived EVs increase the polarization of macrophages *in vitro* into the classic M1 subtype (Wang et al., 2015). M1 macrophages possess pro-inflammatory activity such as phagocytosis and secretion of pro-inflammatory cytokines, whereas the alternatively activated macrophages (M2 macrophages) regulate mostly the resolution phase of inflammation including the repair of the damaged tissues. MiRNAs play a major role in transducing the functional program harbored by EVs (Rayner and Hennessy, 2013; Xu et al., 2013; Arora et al., 2017). They are a class of small non-coding RNAs that negatively regulate gene expression by complementary binding to the 3′ untranslated region (3′ UTR) of their mRNA targets, and either silence translation or decrease mRNA levels. MiRNAs are involved in the regulation of development, differentiation and activation of immune cells (Baumjohann and Ansel, 2013; Montagner et al., 2014; Mehta and Baltimore, 2016). It was shown that uptake of the schistosomal enclosed miRNAs miR-125b and bantam by macrophages expand their proliferation and the production of the M1 cytokine TNF-α (Liu et al., 2019). Decreasing either the number of host monocytes or the TNF-α levels in the infected mice alleviate the worm and egg burden and consequently the pathology. Secreted-EVs from *S. mansoni* schistosomula are also internalized by human monocyte-derived dendritic cells (DCs) and increase their expression of IL-12 (Kuipers et al., 2020), another hallmark cytokine of the M1 response, and a powerful inducer of the Th1 pathway (see below). These data altogether indicate that schistosomal EVs skew the innate immune response toward the M1 pathway, and this deviation is beneficial for the parasite survival.

### Schistosomal EVs Down-Modulate the Adaptive Th2 Immune Response

Pathologically, the acute stage of schistosomiasis begins 1-2 weeks following the cercarial penetration and continues until the adult parasites are set at the blood vessels. The chronic stage starts with the egg deposition and can last for years. The acute infection initiates a strong Th1 reaction, which in mice persists for ~ 5 weeks (Fallon et al., 1998; Pearce et al., 2004), whereas the chronic stage promotes the Th2 response (Fairfax et al., 2012; Maizels et al., 2012).

T helper (Th; CD4^+^) cells have a fundamental role in orchestrating the immune response. After the first encountering of naïve Th cell with the appropriate antigen ^_^ as a pathogen-derived peptide that is presented by antigen presenting cells (APCs; initially DCs and subsequently other APCs such as macrophages) ^_^ it can differentiate toward effector or regulatory lineages (Avni and Rao, 2000; Ansel et al., 2006; Lee et al., 2006; Wilson et al., 2009; Zhou and Littman, 2009; Sallusto, 2016). Each lineage is characterized by the expression of a distinct set of cytokines that eventually instruct the strategy of the immune response. IFN-γ is the signature cytokine of Th1 cells, IL-4, IL-5, and IL-13 (the ‘Th2 cytokines’) of Th2 cells, IL-17 of Th17 cells, and TGF-β and IL-10 of T regulatory (Treg) cells. IL-10 is also expressed by other immune cells such as the M2 macrophages and Th2 cells. IFNγ exerts protective functions during intracellular infections, IL-17 contributes to the host defense against extracellular infections, Th2 cytokines play a major role in response to extracellular parasites, and Treg derived cytokines prevent potential self-reactivity and dampen hyper-immune response (Shamriz et al., 2016; Avni and Koren, 2018). A poorly understood aspect of schistosomiasis is the decline in the Th2 response at the chronic stage after the initial peak at approximately week 8 of infection in humans (Pearce and MacDonald, 2002; Dunne and Cooke, 2005). This decay is intriguing because it occurs even though, without treatment, the parasitic worms live for years and continue to produce eggs (Fairfax et al., 2012; Maizels et al., 2012; Nutman, 2015); There are evidence of infected immigrants that carry schistosomal eggs in their feces decades after leaving the endemic areas (Warren et al., 1974; Hornstein et al., 1990).

Macrophages and DCs incorporate EVs *via* phagocytic or endocytic processes. However, in our quest to understand the decline in the Th2 response during the chronic stage of schistosomiasis, we found that adult *S. mansoni*-secreted-EVs are internalized by Th cells, and downregulate their differentiation toward the Th2 lineage, in an APC-independent manner (Meningher et al., 2020). Inside the Th cells, the schistosomal EV-enclosed miRNAs modulate restrictedly the Th2 transcriptional program, most prominently reduce the expression of the Th2-lineage specifying transcription factor *Gata3* and of the Th2 cytokines *Il-4*, *Il-13*, and *Il-5*. This decrease is not accompanied by alteration in the expression levels of either Th1, Th17 or Treg specifying transcription factors or their hallmark cytokines. *In-vivo*, the schistosomal miRNAs are found in Th cells that are isolated from Peyer’s patches and mesenteric lymph nodes of infected mice. Mechanistically, the schistosomal-enclosed miRNA miR-10 targets MAP3K7 and consequently down-modulates the activity of NF-kB, a critical transcription factor for Th2 cell differentiation and function (Meningher et al., 2020). This is probably only one example, out of many unexplored yet, of anti-Th2 function that is mediated by the EV-derived miRNAs.

The Th2 response, in which Th2 cells are the major players, was evolved during evolution as the most appropriate reaction to multicellular pathogens and tissue injury (Allen and Maizels, 2011; Harris and Loke, 2017). Th2 cells promote macrophage differentiation toward the M2 pathway, and these alternative macrophages can directly bind opsonized parasite larvae through complement components or antibodies to reduce larvae motility (Burke et al., 2009; Maizels et al., 2009; Allen and Maizels, 2011; Neill and McKenzie, 2011; Fairfax et al., 2012; Maizels et al., 2012; Ariyaratne and Finney, 2019). Th2 cytokines recruit also eosinophils that can damage schistosomula through antibody-dependent cell-mediated cytotoxicity (ADCC), which eventually employ degranulation and parasite killing. The humoral profile of Th2 type immunity is associated with elevation levels of the IgG1, IgE and IgG4 isotype antibodies. IgE functions largely through its ability to bind eosinophils and mast cells, whereas IgG4 is more associated with the resolution phase. Therefore, increasing the innate immune response of the M1 pathway and downregulation of the adaptive immune response of the Th2 pathway, reflect the schistosomal efforts to evade hostile environment. However, the relationship between the parasite and its host is more complicated.

### Schistosomal Eggs and the Th2 Double Edged Sword

During the chronic stage, a fraction of the produced eggs is not excreted successfully and become permanently lodged in organs such as the intestine, liver (*S. mansoni* and *S. japonicum)*, bladder, and urogenital system (*S. haematobium)*. These tissue-trapped eggs are actually the major cause to the severe pathology of schistosomiasis (Burke et al., 2009; Fairfax et al., 2012; Kamdem et al., 2018; Ariyaratne and Finney, 2019; Giorgio et al., 2020). Egg antigens, which are taken up by dendritic cells, promote an APC-dependent Th cell differentiation toward the Th2 pathway (Pearce, 2005). Accumulation of the Th2 cells around the trapped eggs increases the infiltration and differentiation of M2 macrophages, eosinophils, and additional immune cells that mediate tissue regeneration and repair and assembly of the Th2-type granulomas. Granulomas are well-defined clusters of inflammatory cells embedded in a collagen-rich extracellular matrix around the parasite eggs to wall off the eggs and their toxic secreted products. Generation of granuloma is Th2 cell-dependent, as T cell-deficient mice or either of the IL-4 or the α-chain of the IL-4R are being unable to mount granulomatous response against schistosomes and are dying earlier than immunologically competent infected mice (Brunet et al., 1997; Fallon et al., 2000). Also, the lack of M2 macrophages reduces the size of the egg-associated granulomas and significantly exacerbated the disease (Herbert et al., 2004; Nascimento et al., 2014). However, continuous repair in chronic Th2 setting results in excessive extracellular matrix deposition, hepatic fibrosis and increased portal hypertension as well as other symptoms that drive the disease morbidity and mortality (Pearce, 2005; Burke et al., 2009; Fairfax et al., 2012; Kamdem et al., 2018; Ariyaratne and Finney, 2019; Mickael and Graham, 2019; Giorgio et al., 2020). The granuloma formation therefore seems to be an evolutionary chosen compromise that allows the host to live with the infection for many years (Hams et al., 2013).

It was shown that in addition to egg-antigens, the *S. japonicum* eggs release EVs that can transfer parasitic miRNA cargo into hepatocytes in the infected mice (Zhu et al., 2016b). The transferred schistosomal miRNA miR-71a attenuates the pathological progression and liver fibrosis (Zhu et al., 2016b), *via* inhibition of the TGF-beta1/SMAD and IL-13/STAT6 pathways by direct targeting of semaphorin 4D (Wang et al., 2020). In addition, treatment of *S. japonicum* infected mice with miR-71a increases the percentage of Treg cells and reduces of the effector Th1/Th2/Th17 cells in the liver. Hence, trapped egg-derived EVs can balance the immune response and tune the fibrosis extent.

Granulomatous response is beneficial to schistosomes by maintaining the host health, but granulomas are also obligatory for the schistosomal reproduction. Intestinal granulomas, which are more organized, aid the transport of the parasite eggs from the mesenteric vasculature to the intestinal lumen and excretion in the feces (Costain et al., 2018; Schwartz and Fallon, 2018; Ariyaratne and Finney, 2019; Giorgio et al., 2020). Although the underlying mechanisms are unclear yet, in mice infected with *S. mansoni* and deficient in the IL-4 expression, almost no eggs were found in the feces (Fallon et al., 2000). The absence of macrophage-specific IL-4Rα signaling, also results in impaired egg expulsion (Herbert et al., 2004). These findings indicate that the Th2-associated granulomas facilitate a non-inflammatory evacuation of the eggs, which is probably the best solution for both humans and schistosomes. It is unclear yet whether the excreted and trapped eggs secrete EVs with alternative cargos.

### The Harbored miRNAs Execute the EV-Functions

**Figure 1:**
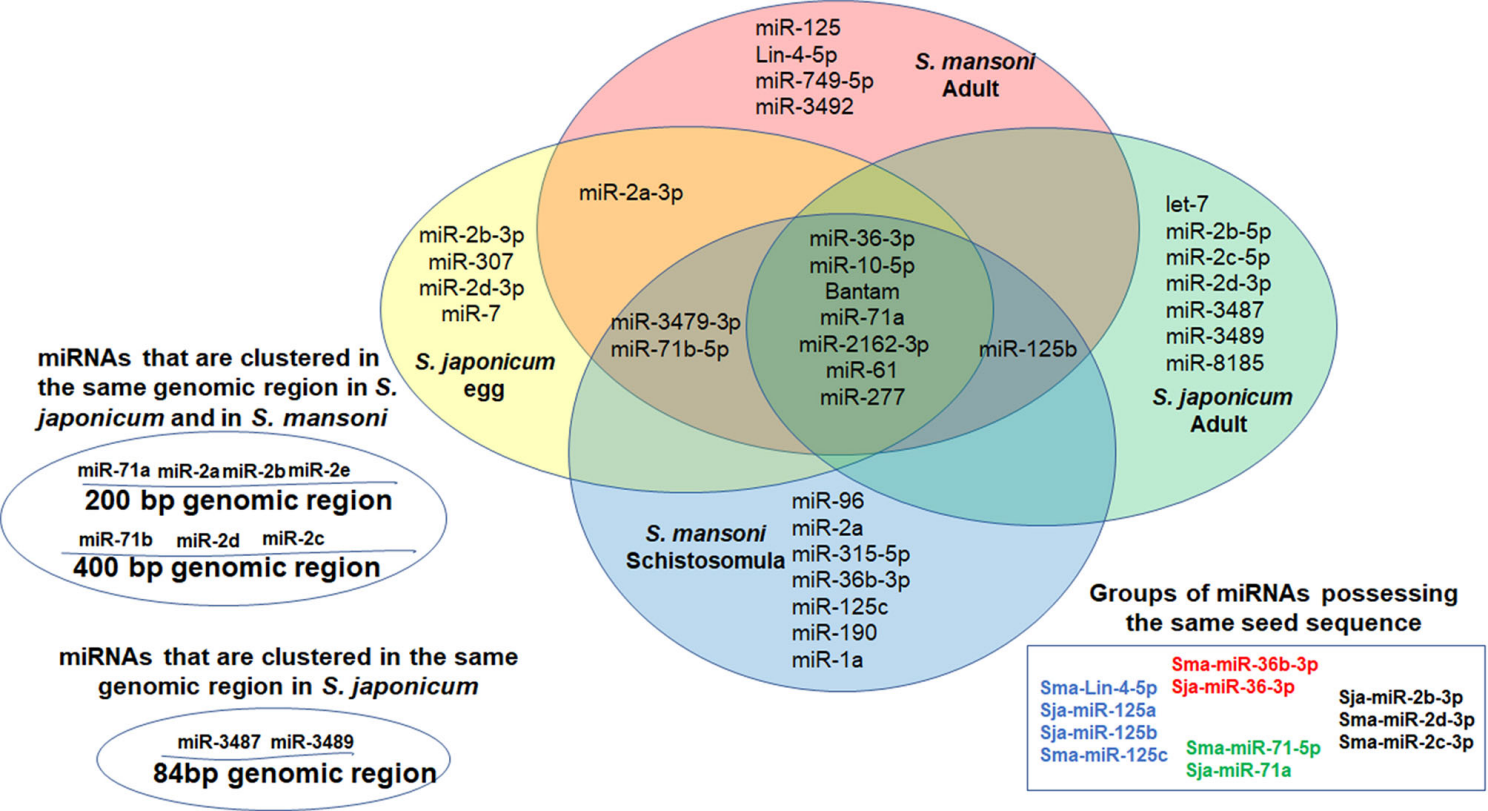
Venn Diagram presenting miRNAs that were found in EVs derived from *S. japonicum* eggs (Zhu et al., 2016b), *S. mansoni* schistosomula (Nowacki et al., 2015) and both *S. japonicum* adult (Zhu et al., 2016a) and *S. mansoni* adult (Samoil et al., 2018). Only miRNAs that are listed in the miRBase and have more than 100 reads are included.

MiRNAs can contribute to quantitative regulation of gene expression, mostly of dosage-sensitive genes for which minor fluctuations in protein expression levels may significantly affect the functional input (Zhu et al., 2014; Arora et al., 2017). In the current miR database (version 21), 79 mature miRNAs of *S. japonicum* and 225 mature miRNAs of *S. mansoni* have been reported (Wang et al., 2010). However, until now, very little is known about the role of the miRNAs in helminthic infections (Zhu et al., 2014; Arora et al., 2017). Since the miRNAs play a major role in the transmission of the EV-instructions, we compared the profiles of EV-harbored miRNAs from different schistosomal stages, according to several recent published studies (**Figure 1**). The ability to perform a reliable quantitative sequencing comparison of EV-contained miRNAs between independent studies is limited, especially due to the absent of standard EV gene. Hence, we have chosen to include only miRNAs that are listed in the miRBase and have more than 100 reads in either *S. japonicum* eggs (Zhu et al., 2016b), *S. mansoni schistosomula* (Nowacki et al., 2015), adult *S. japonicum* (Zhu et al., 2016a) or adult *S. mansoni* (Samoil et al., 2018) (**Figure 1**). The miRNA sequences are presented in **Table 1**. Most of the miRNAs are found in more than one developmental stage. Six of the miRNAs are clustered in two conserved genomic regions in both *S. japonicum* and *S. mansoni*; miR-71a, miR-2a, and miR-2b are clustered in one region and miR-71b, miR-2c, and miR-2d are clustered in another genomic region. MiR-3487 and miR-3489 are clustered in a third genomic region in *S. japonicum* (as shown in **Figure 1**). This clustering may suggest the existence of a local EV-packaging signal (Zhang et al., 2015), although there is no evidence for that in schistosomes. Several miRNAs that are expressed through all stages possess the same seed sequence (**Figure 1**) and therefore putatively regulate the same targets.

**Table 1:** Sequences of miRNAs from schistosomal EVs that were taken from the miRBase.

Table 1a: miRNAs that were found in all EVs		
miRNA	Accession	Sequence
sja-miR-36-3P	MIMAT0016258	5′-c**caccggg**uagacauucauucgc-3′
sja-miR-10-5p	MIMAT0016253	5′-a**acccugu**agacccgaquuugg-3′
Sja-Bantam	MIMAT0010177	5′-u**gagaucg**cgauuaaagcuggu-3′
sja-mir-71a	MIMAT0010176	5′-u**gaaagac**gaugguagugaga-3′
miR-2162-3p	MIMAT0016273	5′-u**auuaugc**aacquuucacucu-3′
sja-miR-61	MIMAT0016259	5′-u**gacuaga**aagugcacucacuu-3′
Sja-miR-277	MI0015296	5′-u**aaaugca**uuuucuggcccg-3
**Table 1b: EVs derived miRNAs with the same seed sequence**		
sma-lin-4-5p	MIMAT0003956	5′-u**cccugag**accuucgacugugu-3′
sja-miR-125a	MIMAT0010178	5′-u**cccugag**acccuuugauuguc-3′
sja-miR-125b	MIMAT0010179	5′-u**cccugag**acugauaauugcuc-3′
sma-miR-125c	MIMAT0033510	5′ -u**cccugag**accuuagaguuguc-3′
sma-miR-36b-3p	MIMAT0033515	5′-c**caccggg**uagacauucauccgc-3′
sja-miR-36-3p	MIMAT0016258	5′-c**caccggg**uagacauucauucgc-3′
sma-miR-71b-5p	MIMAT0025043	5′-u**gaaagac**uugaguagugagacg-3′
sja-mir-71a	MIMAT0010176	5′-u**gaaagac**gaugguagugaga-3′
sja-miR-2b-3p	MIMAT0016248	5′-u**aucacag**cccugcuugggacaca-3′
sma-miR-2d-3p	MIMAT0025035	5′-u**aucacag**uccugcuuagguga-3′
sma-miR-2c-3p	MIMAT0025034	5′-u**aucacag**ccgugcuuaagggc-3′
**Table 1c: EVs derived miRNAs that were found in part of the schistosomal stages**		
miRNA	Accession	Sequence
sja-miR-3479-3p	MIMAT0016275	5′-u**auugcac**uuaccuucgccuug-3′
sja-miR-3487	MIMAT0016296	5′-u**ccucgaa**cuguuguggcca-3′
sja-miR-3489	MIMAT0016298	5′-g**ccacaac**aguucgaggacg-3′
sme-miR-315-5p	MIMAT0011282	5′-u**uuugauu**guugcucugagaguu-3′
sma-miR-190-5p	MIMAT0025027	5′-u**gauaugu**auggguuacuuggug-3′
sma-miR-1a-5p	MIMAT0033630	5′-u**ggaaugu**ggcgaaguaugg-3′
sja-miR-8185	MIMAT0032784	5′-a**ggaucga**ugaacggagcauu-3′
sja-miR-3492	MIMAT0016301	5′-a**uccgug**cugagauuuogucu-3′
sma-miR-96-5p	MIMAT0033635	5′-c**uuggcac**uuuggaauugucac-3′
sme-miR-749	MIMAT0004016	5′-g**cugggau**gagccucgguggu-3′
sja-miR-307	MIMAT0016270	5′-u**cacaacc**uacuugauugag-3′
sja-miR-7-5p	MIMAT0016249	5′-u**ggaagac**uggugauauguuguu-3′
sja-let-7	MIMAT0010175	5′-g**gagguag**uucguuguguggu-3′
sma-miR-2c-5p	MIMAT0025033	5′-u**cccuugu**ucgacugugaugug-3′
sma-miR-2d-5p	MIMAT0032135	5′-g**ucauccu**uggauugugauu-3′
sja-miR-2a-5p	MIMAT0016245	5′-c**agucaau**auuggcugauggca-3'
sja-miR-2b-5p	MIMIAT0016247	5′-c**gucucaa**aggacugugagcca-3′
sja-miR-2a-3p	MIMAT0016246	5′-u**cacagcc**aguauugaugaacg-3′

Bold marks the seed sequences.

Although very little is known about the functions of EV-associated miRNAs (Samoil et al., 2018; Bischofsberger et al., 2020), it is very likely that many of them are involved in the immune evasion. MiR-36-3p, miR-10-5p, miR-71a, miR-2162-3p and miR-61, were found in EVs from all stages of schistosomal development and have a known involvement in immune regulation. MiR-36-3p regulates ERK1/2-induced EMT in pancreatic ductal adenocarcinoma (Hu et al., 2018). ERK1 is also important for Th2 differentiation and development in experimental model of asthma (Goplen et al., 2012). MiR-10-5p downregulates NF-kB, which is critical for Th2 differentiation (Meningher et al., 2020). Bantam induces the differentiation toward the M1 macrophages (Liu et al., 2019), and additional 39 potential human targets (Samoil et al., 2018). MiR-71a attenuates the pathological progression of liver fibrosis (Wang et al., 2020). MiR-2162-3p, a schistosome-specific microRNA that is consistently present in the hepatic stellate cells of *S. japonicum* infected mice, promotes hepatic fibrosis by regulating TGFβ receptor III, a negative regulator of TGF-β signaling (He et al., 2020). MiR-61 targets Vav-1, (Yoo and Greenwald, 2005; Jannot and Simard, 2006), a critical signaling molecules of the immune cells (Katzav, 2015). The miR-125 family appears in EVs from all stages except eggs, and it was shown that miR-125b promotes the M1 pathway (Liu et al., 2019). This initial analysis support the idea that the EV-enclosed miRNAs have a fundamental role in executing the immune modulation by the schistosomes.

### Schistosomal EV-Guidance

In the murine model, the adult *S. mansoni* worms are located at the mesenteric and small intestine venules, which are drained by the gut-associated lymph nodes. The schistosomal miRNAs miR-10 and Bantam were found selectively in the gut-associated lymph nodes, the Peyer’s patches and mesenteric lymph nodes, and not in the inguinal lymph node or spleen (Meningher et al., 2020). These findings suggest a targeted long-distance delivery of the schistosomal EVs through the lymphatic system, rather than a systemic distribution through the blood.

The specificity is probably imposed by membrane associated proteins or other cell surface molecules. It was shown that the uptake of the S*. mansoni* schistosomula-derived EVs by monocyte-derived DCs is mainly mediated *via* the C-type lectin receptor DC-SIGN (CD209) (Kuipers et al., 2020). DC-SIGN is an adhesion molecule that recognizes and binds high-mannose-containing glycoproteins on pathogens, and is present on the surface of both macrophages and dendritic cells.

Studies performed proteomic analysis of schistosome-derived EVs (Nowacki et al., 2015; Sotillo et al., 2016; Zhu et al., 2016a; Samoil et al., 2018; Meningher et al., 2020) are presented in **Table 2**. EV-associated proteins of adult schistosomes include well-described exosomal markers designated in ExoCarta such as heat sock proteins (HSP70), energy-generating enzymes (enolase, pyruvate kinase, GAPDH, phosphoglycerate kinase 1), cytoskeletal proteins (actin, tubulin, fimbrin), and tetraspanins. All of these proteins have already described as the most frequently secreted proteins from *S. japonicum* and *S. mansoni* (Samoil et al., 2018). These proteomic analyses were performed mainly to confirm the characterization of schistosomal vehicle as EV, according to the manually updated online database of EV-proteins, -RNAs and -lipids (Pathan et al., 2019) listed in the Vesiclepedia (http://microvesicles.org/extracellular_vesicle_markers). However, functional studies to explore their function in the targeted delivering and immune modulation are still missing.

**Table 2:** Proteomic analyses of schistosome-derived EVs.

	Mammalian Gene Symbol	Protein name	International Journal for Parasitology 2015 (Javier Sotillo et al.)	Scientific Reports 2016 (Lihui Zhu et al.)	Journal of Extracellular Vesicles 2015 (Fanny C. Nowacki et al.)	EMBO reports 2020 (Tal Meningher et al.)
1	PDCD61P	programmed cell death 6 interacting protein	√		√	√
2	GAPDH	glyceraldehyde-3-phosphate dehydrogenase	√	√	√	√
3	HSPA8	heat shock 70 kDa protein 8	√	√	√	√
4	ACTB	beta Actin,	√	√	√	√
5	ANXA2	annexin A2	√	√	√	√
6	YWHAZ (14-3-3)	tyrosine 3-monooxygenaseltryptophan 5-monooxygenase activation protein,		√	√	√
7	PKM	pyruvate kinase		√		√
8	ENO1	enolase 1,(alpha)		√	√	
9	HSP90AA1	heat shock protein 90kDa alpha		√		
10	tetraspanin	CD9 (SM23), CD63(TSP-2),CD81	(TSP-1,TSP-2, TSP-4, TSP-18)	TSP-1, TSP-(CD63)	TSP-2(CD63)	TSP-2(CD63), SM23
11	EEF1A1	translation elongation factor 1-alpha		√	√	√
12	PGK1	phosphoglycerate kinase		√		√
13	CLTC	clathrin, heavy chain				
14	ALDOA	aldolase	√	√	√	√
15	EEF2	eukaryotic translation elongation	√	√		

### Summary and Perspective

**Figure 2:**
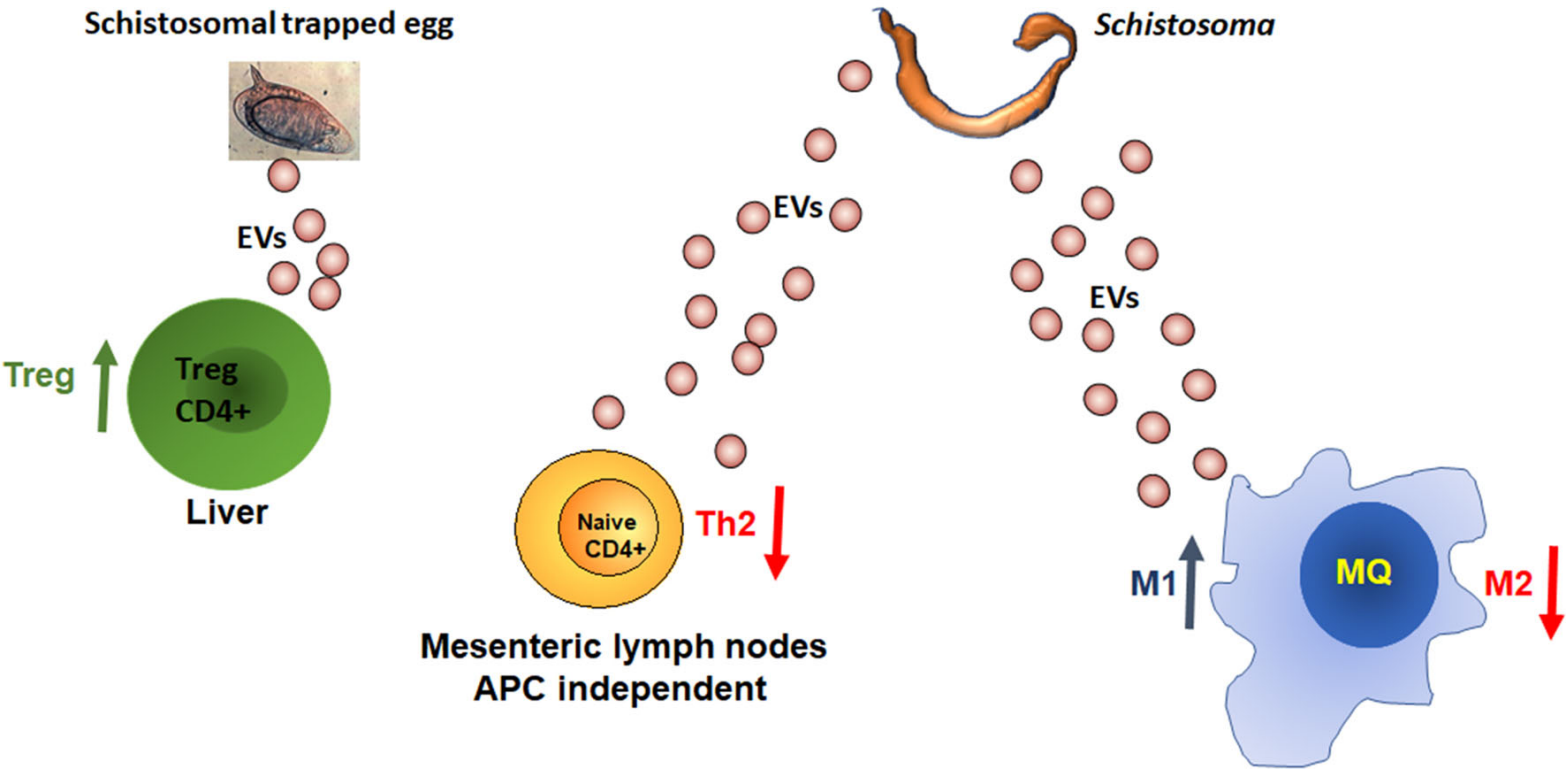
MiRNAs from schistosomal EVs manipulate the host immune response. More details in the text.

Although there are only few studies that characterize the schistosome-secreted EVs, it is possible to conclude that schistosomes from all the developmental stages, including the eggs, secrete EVs. These EVs promote a more permissive immune response to the parasite. The EV-derived miRNAs promote the M1 pathway of the innate immunity and divert the adaptive immunity away from the Th2 pathway (**Figure 2**). However, since the Th2 response is crucial for the host survival and eggs departure, the regulation of the Th2 response by the schistosomes is carefully balanced and targeted, as EV-contained miRNAs from adult schistosomes reduce the differentiation of the Th2 cells selectively in the mesenteric lymph nodes, whereas, miRNA derived from EVs of trapped eggs increases the percentage of Treg cells in the liver and reduces of the effector Th1/Th2/T17 cells. Further studies are required to assess precisely the content, function and targeting of EVs from different developmental stages and physiological circumstances.

From what we have learned so far, the modified immune response by the parasite is in accordance with the ‘hygiene hypothesis‘ (Stiemsma et al., 2015; Versini et al., 2015; Alexandre-Silva et al., 2018; Bach, 2018), associating the dramatic increase in autoimmune and allergic diseases, observed in recent decades in industrialized countries, with the reduced exposure to diverse infectious agents. Indeed, epidemiological evidence supports the fact that schistosomiasis can protect against allergy in an endemic population (Medeiros et al., 2003; Oliveira et al., 2014). Therefore, exploring the schistosomal ‘strategy’ may facilitate the development of novel diagnostic tools (Meningher et al., 2017; Weerakoon et al., 2018; Silva-Moraes et al., 2019), vaccines (Sotillo et al., 2016; Mekonnen et al., 2020) and therapeutic approaches for human schistosomiasis and immune dysregulation (Siles-Lucas et al., 2015; Ayelign et al., 2020).

## Author Contributions

These authors have contributed equally to this work and share senior authorship. All authors contributed to the article and approved the submitted version.

## Conflict of Interest

The authors declare that the research was conducted in the absence of any commercial or financial relationships that could be construed as a potential conflict of interest.
